# Interactions between *Meteorus pulchricornis* and *Spodoptera exigua* Multiple Nucleopolyhedrovirus

**DOI:** 10.1673/031.013.1201

**Published:** 2013-02-15

**Authors:** Hui-Fang Guo, Ji-Chao Fang, Wan-Fang Zhong, Bao-Sheng Liu

**Affiliations:** Institute of Plant Protection, Jiangsu Academy of Agricultural Sciences, Nanjing 210014, China

**Keywords:** biological activity, parasitism, virus transmission

## Abstract

Baculoviruses may interact with parasitoids in the same host. A previous study has shown that infection of larvae with *Spodoptera litura* nucleopolyhedrovirus (SpltNPV) was deleterious to the survival and development of *Meteorus pulchricornis* (Wesmael) (Hymenoptera: Braconidae). In this paper, the interactions between *M. pulchricornis* and *Spodoptera exigua* multiple nucleopolyhedrovirus (SeMNPV) in *Spodoptera exigua* (Hübner) (Lepidoptera: Noctuidae), a permissive host of the virus and parasitoid, were investigated. The results showed that the effect of *M. pulchricornis* on SeMNPV and the effect of the virus on the parasitoid both depended on the concentration of the virus and the interval between viral infection and parasitism. Whether *S. exigua* was treated with the parasitoid and virus simultaneously or 1 day apart, the biological activities of 10^5^, 10^6^, and 10^7^ OBs/mL SeMNPV were all significantly improved by *M. pulchricornis*. In contrast, the biological activity of 10^3^ OBs/mL SeMNPV was significantly decreased when the host was exposed to the virus and parasitoid simultaneously. Regarding the impact of SeMNPV on *M. pulchricornis*, exposing the host to the parasitoid and SeMNPV with concentrations lower than 10^6^ occlusion bodies (OBs)/mL produced no negative effects on the parasitoid. The results also showed that ingestion of SeMNPV by adult stage *M. pulchricornis* significantly increased the number of parasitoid offspring that successfully emerged from the host. Furthermore, *M. pulchricornis* was found to transmit SeMNPV among populations of *S. exigua*. Taken together, these findings indicate that *M. pulchricornis* integrated with an appropriate concentration of SeMNPV has the potential to improve the efficacy of biological control against *S. exigua*.

## Introduction

Parasitoids and baculoviruses are common natural enemies of insect pests, and both are considered major components of biological control against most lepidopteran pests (Moscardi 1999; Cory and Myers 2003; Brodeur and Boivin 2004). To effectively integrate baculoviruses and parasitoids in biological control programs, it is particularly important to understand the interaction between these two types of natural enemies. Much research has investigated the relationship of baculoviruses and parasitoids, and these findings have been reviewed elsewhere (Cossentine 2009). Previous results showed that the interaction between baculoviruses and parasitoids depended on both species and the interval between parasitism and virus infection. Regarding the impact of parasitoids on baculoviruses, the effect may be positive or negative, or there may be no effect at all. In hosts with reduced susceptibility to viruses, parasitism facilitates viral infection. For example, *Manduca sexta* larvae, which are semipermissive hosts of *Autographa* californica multiple nucleopolyhedrovirus (AcMNPV), died more rapidly and at higher levels from AcMNPV*hsp70/lacZ* infection when parasitized by *Cotesia congregata* (Washburn et al. 2000). *Spodoptera littoralis*, another semipermissive host of AcMNPV, was also more susceptible to viral infection when parasitized by *Chelonus inanitus* and its polydnavirus (Rivkin et al. 2006). While in permissive hosts, parasitoids decrease the virulence of baculoviruses. For example, the lethal concentration for 50% of individuals of *Helicoverpa armigera* NPV against cotton bollworm larvae was higher in *Microplitis demolitor*-parasitized *H. armigera* than in non-parasitized hosts (Murray et al. 1995). Decreased virulence of *Agrotis segetum* granulovirus GV was also observed in host larvae that were parasitized by *Apanteles telengai* compared to non-parasitized hosts (Santiago-Alvarez and Caballero 1990). In some virus-parasitoid combinations, however, there is no difference in the virulence of baculovirus in parasitized and non-parasitized hosts. *Spodoptera frugiperda* NPV showed no change in virulence when the host larvae were parasitized by *C. insularis* (Escribano et al. 2001). The efficacy of *Agrotis segetum* granulovirus GV was not affected in larvae of *A. segetum* parasitized by *Campoletis annulatus* (Santiago-Alvarez and Caballero 1990). *C. insularis*-parasitized and non-parasitized larvae also exhibited similar quantitative responses to a challenge of virus inoculum (Escribano et al. 2000b). The impact of baculoviruses on parasitoids should also be considered. Although parasitoids are not susceptible to viral infection, their size and survival are often dependent on the quality or survival of the virus-infected host. More specifically, parasitoid survival is largely dependent on the time of virus inoculation and the dose of virus. The survival of *M. gyrator* within a *Lacanobia oleracea* (L.) granulovirus (LoGV)-infected host increased as the interval between parasitism and infection increased, and no parasitoids emerged from hosts infected with *Lacanobia oleracea* (L.) granulovirus before parasitoid oviposition (Matthews et al. 2004). The parasitoids *M. demolitor, Cotesia kazak*, and *Hyposoter didymator* required a time advantage of 3 days before the host was exposed to NPV to successfully complete their development (Murray et al. 1995). The inoculation dose of *Mamestra brassicae* NPV and the timing of parasitoid release also had significant effects on the development of *Habrobracon hebetor* in virus-infected *Spodoptera exigua* (Hübner) (Lepidoptera: Noctuidae) (Rabie et al. 2010).

*Meteorus* pulchricornis (Wesmael) (Hymenoptera: Braconidae), the solitary egg-larval endoparasitoid, is an important natural enemy of many major insect pests, including *S. litura, H. armigera, Plutella xylostella*, and *M. brassicae* (Takashino et al. 1998). It is the most abundant parasitoid of *S. exigua* in Nanjing, Jiangsu Province in China (Liu and Li 2006). The research on the interactions between *M. pulchricornis* and *S. litura* indicate that the survival of the parasitoid in virus-infected larvae is dependent on the interval between parasitism and viral infection and the inoculation dose of SlNPV. *M. pulchricornis* was adversely affected by SlNPV when the host larvae were exposed to high doses of virus 1 and 3 days post-parasitization (Nguyen et al. 2005). The beet army worm, *S. exigua*, is a polyphagous pest of numerous cultivated crops, including cabbage, cotton, tomato, celery, lettuce, and alfalfa, and it is widely distributed in Asia, Europe, North America, and Africa. *S. exigua* multiple nucleopolyhedrovirus (SeMNPV) has been exploited as a biological control agent. It has been reported that the parasitoid *Microplitis pallidipes* combined with SeMNPV could more effectively suppress *S. exigua* than the parasitoid alone (Jiang et al. 2011).

The aim of this study was to clarify the interactions between *M. pulchricornis* and SeMNPV in *S. exigua*. We investigated the effects of *M. pulchricornis* parasitism on the virulence and killing speed of SeMNPV, the effect of viral infection on the survival and development of the parasitoid, and the transmission of SeMNPV by *M. pulchricornis*. These findings may help the development of methods that integrate parasitism and baculovirus infection for effective control of *S. exigua*.

## Materials and Methods

### Insects

Individual *S. exigua* were collected from cabbage fields in Nanjing, Jiangsu Province, China, and reared in the laboratory at 28±1° C, 50–60% RH, and with a 14:10 L:D photoperiod. They were maintained on an artificial diet for 25 generations. Groups of larvae were reared on an artificial diet in jars (diameter 30 cm, height 20 cm) up to the third instar stage of development. Larvae were then reared individually in 6-well tissue culture plates. Groups of pupae were maintained in jars, as above, and adults were paired in cages (30 cm × 30 cm × 30 cm) and fed 5% (W/V) sugar solution. Eggs were laid on paper sterilized with 5% (w/w) formaldehyde solution, and hatched larvae were reared until the desired developmental stages.

The *M. pulchricornis* was donated by Prof. Li Baoping of Nanjing Agricultural University, and it originated from parasitized & *exigua* larvae collected in soybean fields in Jiangpu, Nanjing, China in 2002. The parasitoids were continuously maintained on *S. exigua* larvae in the laboratory, and all were unfertilized females, which produce only female broods. Twenty to thirty adult parasitoids were reared in a glass bottle (15 cm height × 12 cm diameter). They were fed 10% honey solution and given *S. exigua* for parasitism. Colonies were maintained at 28° C with a 14:10 L:D photoperiod.

### Virus preparation

Isolates of SeMNPV were originally collected from infected *S. exigua* in Nanjing, China. The SeMNPV had been propagated in *S. exigua* for more than 20 generations. Occlusion bodies (OBs) were obtained by feeding artificial diets contaminated with the virus to third instar larvae in 6-well tissue culture plates. The infected larvae were reared at 28° C, 50% RH, and with a 14:10 L:D photoperiod, then collected after death. To produce sufficient viruses for the experiments, OBs were recovered from pooled batches of infected larvae and purified. The purification process involved filtration through cheesecloth to remove larval debris, and centrifugation at 500 rpm for 5 min and 3000 rpm for 15 min. The purified OBs were resuspended in distilled water and quantified with a haemocytometer. Five counts per haemocytometer and three sub-samples per suspension were measured to reduce counting and dilution errors. The viral suspensions were stored at 4° C until use.

### Susceptibility of parasitized host larvae and parasitoid survival in infected hosts

Bioassays were performed on newly moulted third instar *S. exigua* larvae that were subjected to the following treatments: exposure to *M. pulchricornis* for 1 day followed by exposure to SeMNPV, simultaneous *M. pulchricornis* and SeMNPV exposure, SeMNPV exposure only or *M. pulchricornis* exposure only. Five different concentrations of virus were administered to larvae 10^3^, 10^4^, 10^5^, 10^6^ and 10^7^ OBs/mL), and 10 µl of each viral suspension was applied to a small pellet (8 mm^3^) of artificial diet. For bioassays on larvae exposed to *M. pulchricornis* and virus simultaneously, 20 artificial diet pellets and 10 µl of viral solution per pellet were placed in a plastic cup. Five *M. pulchricornis* 3-day-old adults and 20 third instar *S. exigua* larvae were placed in the same cup for 1 day. The cups were maintained in incubators at 28° C, 50% RH, and with a 14:10 L:D photoperiod. *S. exigua* larvae were then individually placed into the wells of 6- well tissue culture plates and fed a fresh, untreated artificial diet. The parasitoids were discarded. Larvae exposed only to *M. pulchricornis* or SeMNPV were subjected to the same treatments. The control larvae were fed a diet with 10 µl sterilized distilled water. All tests were replicated 3 times and 20 larvae were used for each replicate.

For bioassays on larvae exposed to *M. pulchricornis* for 1 day then exposed to SeMNPV, 5 *M. pulchricornis* 3-day-old adults and 20 *S. exigua* third instar larvae were placed in the same cup simultaneously for 1 day. & *exigua* larvae were then individually placed into the wells of 6-well tissue culture plates and fed a small 8 mm^3^ pellet of artificial diet with 10 µl of viral solution. After 24 hrs, the larvae that consumed the contaminated diet were maintained and fed a fresh, artificial diet without virus. Bioassays were also conducted on larvae exposed to only *M. pulchricornis* for 1 day or SeMNPV simultaneously. These experiments were maintained in incubators at 29° C, 50% RH, and with a 14:10 L:D photoperiod. The survival of *S. exigua* larvae and parasitoids were checked daily until no new dead larvae and no offspring were observed. The parasitoids were kept for further assessment of SeMNPV transmission.

### Viral transmission of SeMNPV by *M. pulchricornis*

The transmission of SeMNPV was investigated in *M. pulchricornis* fed a honey solution containing SeMNPV. Specifically, 6 newly emerged *M. pulchricornis* were fed a mixture of 10^8^ OBs/mL SeMNPV and 10% honey solution in a cup with a piece of filter paper containing the solution. The control parasitoids were fed a 10% honey solution without virus. After 1 day, the parasitoids were transferred to individual cups and fed honey solution without NPV, and 20 *S. exigua* third instar larvae were placed in each cup for parasitism. Each day, the *S. exigua* larvae were moved to 6-well tissue culture plates and replaced with a new batch of 20 third instar unparasitized larvae. The virus-induced larval mortality and the emergence of parasitoids were recorded every 24 hrs until larvae died or pupated. The experiments were repeated 3 times.

### Statistical methods

All data, namely mortality, the time to death, percentage of parasitism, and developmental time, were analyzed using a one-way analysis of variance, and means were compared using Tukey's Standardized range honestly significant difference test (α = 0.05).

## Results

### Effects of *M. pulchricornis* parasitism on SeMNPV pathogenesis in *S. exigua*

**Figure 1.**
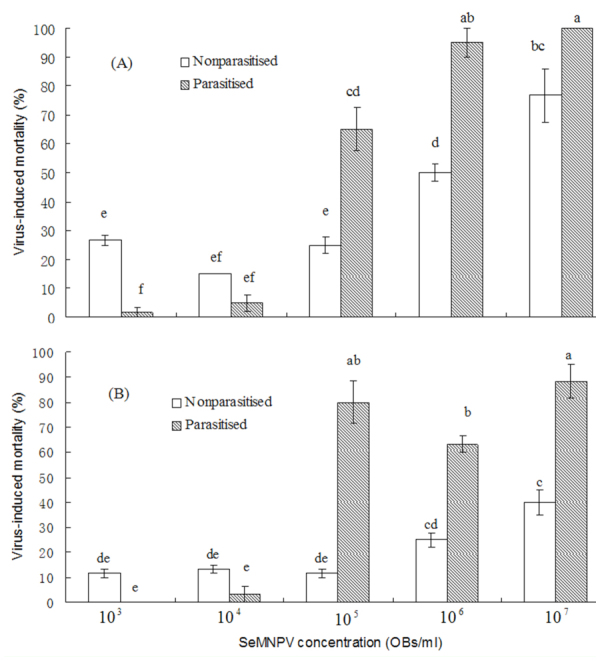
Virus-induced mortality of parasitized and nonparasitized *Spodoptera exigua* inoculated orally with varying concentrations of SeMNPV. (A) shows the effect on larvae exposed to *Meteorus pulchricornis* and SeMNPV simultaneously, and (B) shows the effect on larvae exposed to SeMNPV following parasitization by *M. pulchricornis* for I day. Each column represents the average of results from three bioassays, with 20 insects used in each bioassay. Columns with the same letters are not significantly different at the 0.05 level. Bars represent standard error. High quality figures are available online. parasitized and non-parasitized larvae. Viral infectivity of parasitized and non-parasitized larvae all increased with increasing concentrations of SeMNPV.

The susceptibilities of *S. exigua* larvae to SeMNPV with prior or simultaneous exposure to *M. pulchricornis* were compared (Figure 1). For larvae exposed to SeMNPV and *M. pulchricornis* simultaneously, differences in virus-induced mortality between parasitized and non-parasitized larvae varied with the concentration of SeMNPV (F = 66.981, df = 9,29, *p* < 0.0001). Parasitism increased susceptibility to the virus at concentrations of 10^5^, 10^6^, and 10^7^ OBs/mL SeMNPV, and the virus-induced mortality of parasitized *S. exigua* third instar larvae was significantly higher than that of non-parasitized larvae. Whereas parasitism decreased viral susceptibility at a concentration of 10^3^ OBs/L SeMNPV, parasitized larvae were less susceptible to SeMNPV than were nonparasitized larvae. At a concentration of 10^4^ OBs/mL SeMNPV, there were no significant differences in viral susceptibility between For larvae exposed to SeMNPV following parasitism for 1 day, susceptibility to viral infection between parasitized and nonparasitized larvae also varied with virus concentration (F = 57.973, df = 9,29, *p* < 0.0001). At SeMNPV concentrations of 10^5^, 10^6^, and 10^7^ OBs/mL, parasitized larvae died at higher levels than non-parasitized larvae. At SeMNPV concentrations of 10^3^ and 10^4^ OBs/mL, viral susceptibility was not significantly different between parasitized and non-parasitized larvae.

**Figure 2.**
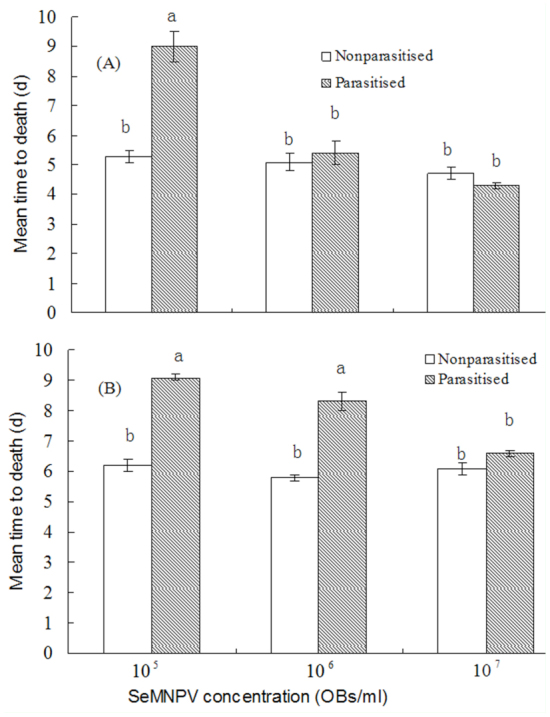
Mean time to death of non-parasitized and parasitized *Spodoptera exigua* inoculated orally with varying concentrations of SeMNPV. Each column represents the average for all larvae killed by the virus in each treatment. High quality figures are available online.

The effect of parasitism on the killing speed of viral infection also revealed dependence on the concentration of SeMNPV and the interval between parasitism and viral infection (Figure 2). For treatment without a delay interval between SeMNPV and *M. pulchricornis* exposure, parasitized *S. exigua* died more slowly than non-parasitized controls at a concentration of 10^5^ OBs/mL SeMNPV. The mean time to death induced by viral infection for parasitized larvae was higher than that of non-parasitized larvae, but the values for the two groups were not significantly different at either 10^6^ or 10^7^ OBs/mL SeMNPV (F = 29.652, df = 5, 17, *p* < 0.0001). For the treatment with a 1 day interval between SeMNPV and *M. pulchricornis* exposure, there was a significant difference in mean time to death between parasitized and nonparasitized larvae at a concentration of 10^5^ and 10^6^ OBs/mL SeMNPV. There were no significant differences observed at 10^7^ OBs/mL SeMNPV (F = 45.533, df = 5, 17, *p* < 0.0001).

**Figure 3.**
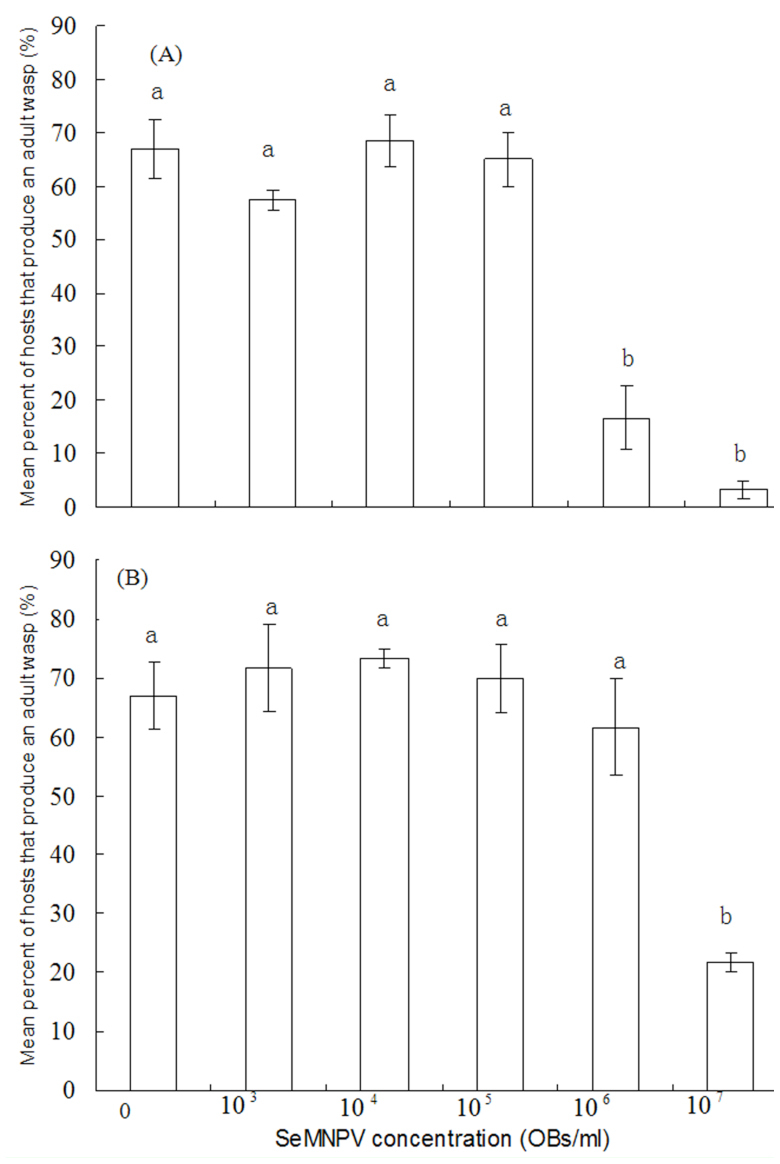
Percent of hosts that produce an adult wasp of virus-inoculated and healthy larvae at various SeMNPV concentrations. Each column represents the average of results from three bioassays, with 20 insects used in each bioassay. High quality figures are available online

### Effects of SeMNPV infection on emergence of *M. pulchricornis* in *S. exigua*

The impact of viral infection on parasitoid emergence depended on the concentration of SeMNPV (Figure 3). When larvae were simultaneously treated with parasitoid and virus, no significant differences were observed in the percentages of successful parasitism between healthy and virus-inoculated larvae at concentrations of 10^3^, 10^4^, and 10^5^ OBs/mL SeMNPV. In contrast, a few *M. pulchricornis* survived in *S. exigua* larvae that were inoculated with 10^6^ and 10^7^ OBs/mL SeMNPV (F = 40.252, df = 5,17, *p* < 0.0001). When larvae were treated with SeMNPV following parasitisation for 1 day, the percentage of successful parasitism in virus-infected larvae was significantly lower than that in healthy larvae, but only at a concentration of 10^7^ OBs/mL SeMNPV. At other concentrations, no differences in successful parasitism in virus-infected and healthy larvae were observed (F = 12.025, df = 5, 17, *p* = 0.0002).

**Figure 4.**
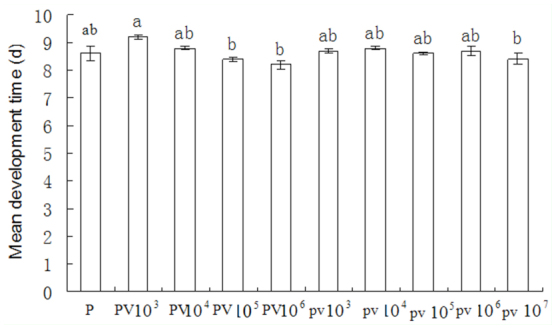
Development time of *Meteorus pulchricornis* that emerged from virus-inoculated and healthy larvae at various SeMNPV concentrations. PV refers to exposure of larvae to *M. pulchricornis* and virus simultaneously, and pv refers to exposure of larvae to *M. pulchricornis* and exposure to SeMNPV I day later. P refers to exposure of larvae to *M. pulchricornis* only. Each column represents the average value for all parasitoids that successfully emerged from the host in each treatment. High quality figures are available online.

The development time (the period from oviposition to cocoon formation) of *M. pulchricornis* was not affected by viral infection (Figure 4). Whether there was a 1 day interval or no interval between parasitism and viral infection, the mean development period (egg to pupa) of parasitoids that emerged from virus-infected larvae ranged from 8.2 days to 9.2 days, which was not different from uninfected controls, but there was significant difference among different viral infections (F = 4.297, df = 9,29, *p* = 0.0032).

### Transmission of SeMNPV by *M. pulchricornis*

**Figure 5.**
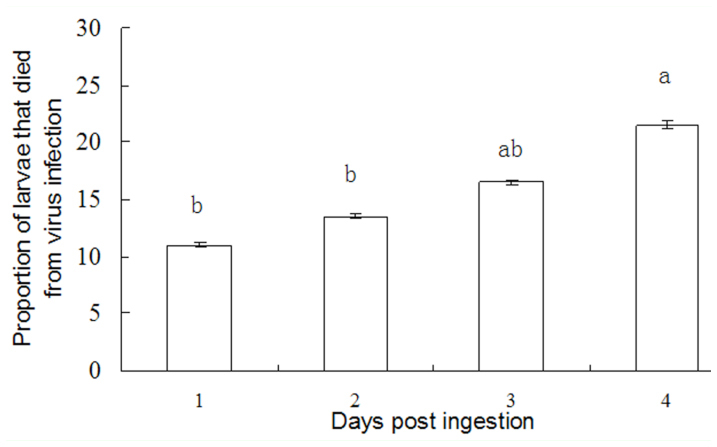
Transmission of SeMNPV by *Meteorus pulchricornis* fed a honey solution containing the virus. Each column represents the average of results from three bioassays using 6 parasitoids per bioassay. High quality figures are available online.

*M. pulchricornis* could transmit SeMNPV horizontally (Figure 5). After parasitoids fed on a mixture of SeMNPV and honey solution for 1 day, they were subsequently transferred and used to treat non-parasitized *S. exigua* larvae for 4 days. One parasitoid could successfully transmit the virus to 21.5% of larvae (died of viral infection) at 4 days postinfection. This result was significantly higher than transmission observed at 1, 2, and 3 days post-infection (F = 13.597, df = 3, 11, *p* = 0.0017).

**Figure 6.**
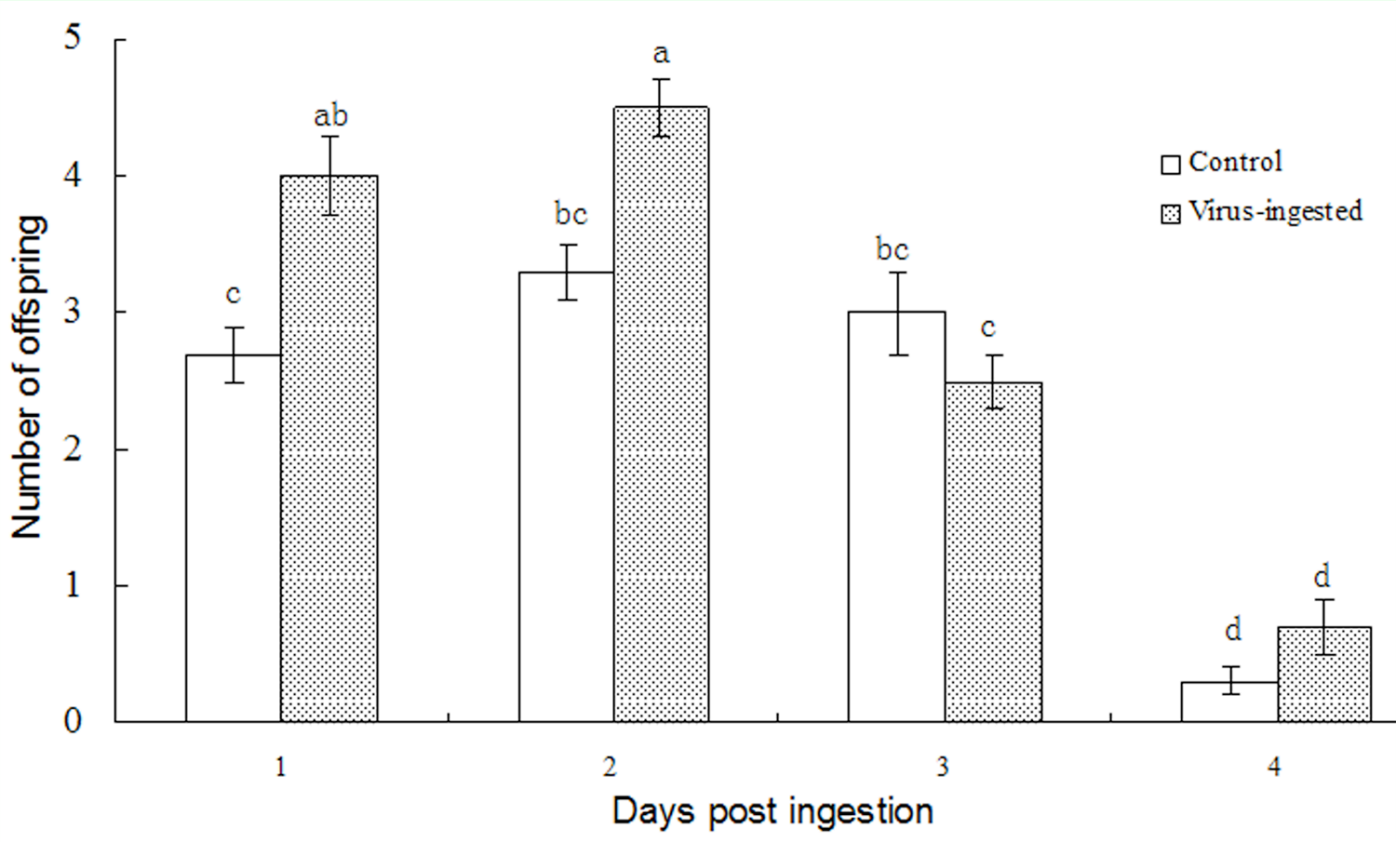
Number of offspring that successfully emerged from the host, produced by *Meteorus pulchricornis* fed a honey solution containing the virus. Each column represents the average of results from three bioassays using 6 parasitoids per bioassay. High quality figures are available online.

Ingestion of SeMNPV by adult-stage *M. pulchricornis* also affected the successful emergence of parasitoid offspring (Figure 6); 1 and 2 days post-ingestion, the average number of offspring that emerged from the host, per parasitoid that ingested the virus, was significantly higher than the control average. No significant difference in the number of cocoons was observed at 3 and 4 days post-ingestion (F = 51.364, df = 7,23, *p* < 0.0001) (Figure 6).

## Discussion

Insect parasitoids and baculoviruses are important for the biological control of insects, and previous studies indicate that the interaction between the two parasites is variable. In some cases, parasitism improves viral susceptibility (Washburn et al. 2000; Rivkin et al. 2006), while in other cases, parasitoids and viruses compete within common hosts, and parasitism decreases viral susceptibility (Santiago-Alvarez and Caballero 1990; Murray et al. 1995). In systems unlike either of these, virus susceptibility is unaffected by parasitism (Hochberg 1991b). Though increased susceptibility is the ideal outcome for biological control, it appears to be less common than the other responses, especially in permissive hosts.

In the present study, the effect of *M. pulchricornis* on the virus was found to vary with the concentration of SeMNPV. When *S. exigua* was exposed to the parasitoid and an appropriate concentration of virus simultaneously or after a 1 day interval, parasitism could increase host susceptibility to SeMNPV. These findings were not similar to results from a previous study investigating the interaction of *M. demolitor* and NPV in *H. armigera*, a permissive host (Murray et al. 1995).

It was also observed in the present study that *M. pulchricornis* parasitism increased the time to virus-induced death compared to nonparasitized larvae. This observation differed from findings reported in *Heliothis zea* that *M. croceipes* increased the killing speed of HzMNPV (Eller et al. 1988). Though the time to virus-induced death was increased by the parasitoid, the dead parasitized larvae were much smaller than the non-parasitized larvae, indicating that *M. pulchricornis* might adversely affect the production of SeMNPV in *S. exigua.* It has been reported that the quantity of . *Spodoptera frugiperda* NPV produced in parasitized and infected *S. frugiperda* larvae was actually less than that in virus-infected larvae, but *C. sonorensis* had no negative effects on the biological activity of the virus (Escribano et al. 2000a).

The effect of SeMNPV on *M. pulchricornis* was dependent on the concentration of the virus and the interval between parasitism and viral infection. Virus inoculation at relatively low concentrations had no negative effect on parasitism, including emergence rate and development time. At higher concentrations, however, the virus had deleterious effects on the parasitoid, with fewer parasitoids emerging from virus-infected larvae. In *S. litura*, the deleterious effects of SpltNPV infection were found to be dependent on the interval between parasitization and viral infection, as well as the inoculation dose of SpltNPV. Postponing the exposure of parasitized larvae to baculovirus increased the percentage of successful parasitoid development, and SpltNPV had no effect on parasitism on the fifth day post-parasitization (Nguyen et al. 2005). The results of the present study showed that the development time of the *M. pulchricornis* that emerged from SeMNPV-infected *S. exigua* was not affected by the virus. Previous reports, on the other hand, found that the development time of *C. sonorensis* was significantly reduced in virus-infected hosts compared to conspecifics that developed in healthy hosts (Escribano et al. 2000b), and that of *M. croceipes* was also shortened in AcMNPV-infected *H. virescens* (McCutchen et al. 1996).

Premature death of parasitoids in virusinfected hosts is thought to be the main reason for the negative effect of viruses on parasitoids. When there is either no interval or an insufficient interval between parasitism and virus inoculation, the number of eggs produced by parasitoids is unaffected, but parasitoids cannot complete development because the larvae die of viral infection quickly, and fewer parasitoids emerge. Premature host death is the most common consequence of host-parasitoid-virus interactions (Eller et al. 1988; Hochberg 1991b). In MbMNPV-infected *S. exigua*, however, the mortality of *H. hebetor* was not due to the death of the host before the parasitoids could complete development (Rabie et al. 2010). During the first 4 days post-infection, there was no significant difference in the number of eggs laid by *M. pulchricornis* in SpltNPV-infected and non-infected *S. litura* larvae. At 5 days post-infection, however, the parasitoid could discriminate non-infected and virus-infected larvae and laid significantly more eggs in noninfected larvae than in virus-infected larvae (Nguyen et al. 2005). In the current experiment, when *S. exigua* larvae were exposed to *M. pulchricornis* and relatively low concentrations of SeMNPV simultaneously or after a 1-day interval, parasitoid survival was not affected by the virus. Hosts exposed to the virus and parasitoid died more slowly than those exposed to the virus only, allowing *M. pulchricornis* to complete development. At higher concentrations of SeMNPV, parasitoid emergence was adversely affected by the virus, as parasitism did not retard virus-induced death. This result is similar to previously reported findings for high doses of virus in other virus-parasitoid interactions (Eller et al. 1988; Nguyen et al. 2005).

Besides interacting with viruses in the same host, parasitoids also have the ability to physically transfer baculoviruses between hosts (Caballero et al. 1991; Hochberg 1991a; Matthews et al. 2004). Females of *Apanteles telengai, Aleiodes gasteratus*, and *Campoletis annulata* that previously oviposited in GV-infected *A. segetum* larvae could transmit the virus to healthy larvae in subsequent ovipositions (Caballero et al. 1991). *Microplitis pallidipes* that ingested SeMNPV in adult stage could transmit the virus to healthy *S. exigua* (Jiang et al. 2011). In our study, *M. pulchricornis* was found to transmit SeMNPV to *S. exigua*. Some larvae died of viral infection, albeit at low levels, after exposure to the virus. The actual virus transmission ability of *M. pulchricornis* might be greater than what has been shown here, as it is likely that more hosts might have been infected with NPV transmitted by parasitoids, but the dose of the virus was too low to cause death. How did the parasitoids transmit virus horizontally? Our, parasitoids were fed on honey solution containing SeMNPV before exposure to host, so they might have not only ingested the virus, but also contaminated their bodies or ovipositors with virus. One possible path for virus transmission is that, after the parasitoids are exposed to hosts with the diet, the virus might contaminate the diet, and then hosts ingest virus-contaminated diet. The other possible path is that the virus-contaminated ovipositor of the parasitoids injects the virus to the hemolymph of the host directly while they produce eggs in hosts. Whether the above is true needs further investigation. We found that ingestion of SeMNPV by adult stage *M. pulchricornis* significantly increased the number of parasitoid offspring that successfully emerged from the host.

A few field or semi-field trials have shown that some parasitoids appear to have positive effects on viral efficacy and host suppression. For example, *M. gyrator* was shown to discriminate between healthy and *Lacanobia oleracea* (L.) granulovirus -infected *L. oleracea* larvae in greenhouse conditions, and the braconid could mechanically transmit the virus at low levels and decrease pest damage on tomato (Matthews et al. 2004). *Microplitis pallidipes* carrying SeMNPV caused a greater *S. exigua* population reduction in greenhouse (Jiang et al. 2011). *Ametadoria misella* oviposited more often in healthy than in HbGV-infected host larvae (Stark et al. 1999). The results of our study support the feasibility of integrating appropriate concentrations of SeMNPV with *M. pulchricornis* for improving the biological control of *S. exigua* in the field.

## Abbreviations

**AcMNPV**,: *Autographe californica* multiple nucleopolyhedrovirus
**OBs**,: occlusion bodies
**SeMNPV**,: *Spodoptera exigua* multiple nucleopolyhedrovirus
**SpltNPV**,: *Spodoptera litura* nucleopolyhedrovirus
